# Impact of COVID-19 Restrictions on Western Australian Children’s Physical Activity and Screen Time

**DOI:** 10.3390/ijerph18052583

**Published:** 2021-03-05

**Authors:** Andrea Nathan, Phoebe George, Michelle Ng, Elizabeth Wenden, Pulan Bai, Zino Phiri, Hayley Christian

**Affiliations:** 1 Telethon Kids Institute, Nedlands, WA 6009, Australia; phoebe.george@telethonkids.org.au (P.G.); michelle.ng@telethonkids.org.au (M.N.); elizabeth.wenden@telethonkids.org.au (E.W.); pulan.bai@telethonkids.org.au (P.B.); zino.phiri@telethonkids.org.au (Z.P.); hayley.christian@uwa.edu.au (H.C.); 2 School of Population and Global Health, The University of Western Australia, Crawley, WA 6009, Australia

**Keywords:** COVID-19, pandemic, physical distancing, children, physical activity, screen time

## Abstract

Physical activity is essential for children’s healthy development, yet COVID-19 physical distancing restrictions such as school closures and staying at home, playground closures, and the cancelling of organised community sport have dramatically altered children’s opportunities to be physically active. This study describes changes in levels of physical activity and screen time from February 2020 (i.e., before COVID-19 restrictions were introduced in Western Australia) to May 2020 (i.e., when COVID-19 restrictions were in place). Parents of children aged 5 to 9 years from Western Australia were eligible to participate and recruited through convenience sampling. An online survey instrument that included validated measures of their children’s physical activity (unstructured, organized, home-based, indoor/outdoor active play, dog play/walking), sociodemographic, and other potential confounders was administered to parents. Paired *t*-tests and mixed ANOVA models assessed changes in physical activity outcomes. The analytic sample comprised parents of 157 children who were 6.9 years of age (SD = 1.7) on average. Overall, weekly minutes of total physical activity (PA) did not change from before to during COVID-19. However, frequency and duration (total and home-based) of unstructured physical activity significantly increased. Outdoor play in the yard or street around the house, outdoor play in the park or playground or outdoor recreation area, and active indoor play at home all significantly increased. Frequency and total duration of organised physical activity significantly declined during COVID-19 distancing. During Western Australian COVID-19 restrictions, there was an increase in young children’s unstructured physical activity and outdoor play and a decrease in organised physical activity. It remains to be seen whether children’s increased physical activity has been sustained with the easing of physical distancing restrictions.

## 1. Introduction

Regular physical activity (PA) is essential for children’s healthy development [1]. For children, the health benefits of PA include maintaining a healthy weight, good mental health, good muscle and bone health, as well as improved motor, cognitive, emotional, and psychosocial development [1]. Furthermore, children who are active when they are young are more likely to continue being active into adulthood and throughout the life course [2]. However, Australian children are barely passing (a D–) when it comes to getting the required physical activity each day [3]. Overall PA encapsulates many types of behaviours. Organised PA (e.g., structured team sports, swimming lessons, dance classes) and unstructured PA (e.g., active commuting to school, walking, riding, active play with friends) contribute in distinct and complementary ways to children’s health. A combination of both organised and unstructured PA confers the greatest benefit to children [4]. However, over the last few decades, children’s time has been increasingly scheduled which has resulted in limited opportunities for children to engage in unstructured PA and free play [5,6]. PA, sedentary behaviour, (e.g., screen time) and sleep are behaviours representing the 24-h movement spectrum, as outlined in World Health Organisation (WHO) and country-specific 24-h movement guidelines for children [7,8]. Therefore, it is important to understand PA behaviour changes alongside changes in screen time and sleep.

Socioecological models of health behaviours emphasise the importance of multiple levels of individual, social, and environmental factors influencing PA behaviours [9]. For children, one of the strongest determinants of young children’s PA is the time they spend outdoors [10]. Other key modifiable factors consistently associated with children’s PA include parent support, access to programs and facilities, and having opportunities to be active [10].

The WHO declared COVID-19 a pandemic on 11 March 2020 [11]. In Australia, the Federal and State Governments worked together implementing restrictions at a national and local level. On 15 March 2020, Western Australia was declared a state of emergency. Two weeks later (30 March 2020), the Federal Government advised gyms and indoor sporting facilities, playgrounds, skate parks, and outside gyms in public places were closed. Australians were told to stay home unless shopping for food and necessities, they had medical or healthcare needs, to exercise—in compliance with the public gathering requirements, or for work or study. It is important to consider the impact of the closure of these facilities, as the cancelling of organised sports and playground closures could significantly impact children’s PA.

The closure and reopening of schools varied across Australian states and territories. From 26 March to 9 April 2020, families in Western Australia were encouraged to keep children at home, if they could access online or other resources to continue their child’s education [12]. School holidays ran from 10–28 April 2020. A cautious and safe reopening of all government schools was announced for students from 29 April, and from 18 May 2020 all Western Australian school students were required to return to school [12]. The reopening of Catholic and independent schools was slightly different and varied on a school-by-school basis.

It is believed that common forms of children’s unstructured PA (e.g., active commuting to school, recess and lunch time, active play in playgrounds and parks) and structured PA (e.g., organised sports, dance, and physical education classes) were affected by COVID-19 restrictions [13]. So far, there is little evidence on the effects of COVID-19 and the associated school closures and physical distancing rules on children’s PA and associated behaviours [14,15,16]. It is likely that structured PA, such as organised sports, declined during this period but it is unknown what impact COVID-19 had on children’s unstructured PA and overall PA levels. There is preliminary evidence that children’s PA decreased as a result of COVID-19 [15,16]. For example, from before to during the pandemic, a 435 min/week reduction in time spent in PA was reported among Chinese children and adolescents, as well as an average 280 min/week increase in leisure screen time [16]. In Canada, children were less active, played outside less, engaged in more recreational screen time, and slept more during COVID-19 restrictions ^16^. It is unknown how children’s PA behaviour changes as a result of COVID-19 have been impacted by common individual (e.g., child age and sex, parent education) and environmental (e.g., access to green space) correlates of children’s PA.

While there is some evidence of the negative impact of COVID-19 on children’s PA, studies are limited and have focused on large age ranges of children [14,15,16]. Western Australia provides a unique context in which to investigate the effects of COVID-19, particularly because there was no sustained community transmission of COVID-19 for nine months [17]. Western Australia is also a relatively isolated Australian state, has high levels of urban sprawl [18], and has enforced the strongest COVID-19 border controls in Australia.

Given the unique characteristics of the Western Australian community, it is important to explore the impact of COVID-19 on children’s PA with home environment factors, as neighbourhood environment attributes have been reported to impact on children’s PA [19]. The aim of this study was to investigate the impact of COVID-19 restrictions on Western Australian young children’s (5 to 9 years) PA behaviour and associated movement behaviours. More specifically, it sought to describe changes in their levels of PA behaviours from February 2020 (i.e., before COVID-19 restrictions were introduced) to May 2020 (i.e., when COVID-19 restrictions were in place) and examine if behaviour changes differed by demographic, social, and home environment factors. Assessing how children’s PA changed during this time can assist with identifying where children and families need support should subsequent COVID-19 waves occur. In addition, information on whether PA changes are sustained and the impact of multiple levels of influence on children’s PA during COVID-19 restrictions will guide future strategies and supports.

## 2. Materials and Methods

This was a retrospective cohort study conducted from 15 May to 5 June 2020. The study was approved by the Human Research Ethics Committee at The University of Western Australia (RA/4/1/7417).

### 2.1. Recruitment and Data Collection

Parents of children aged 5 to 9 years who lived in Western Australia were eligible to participate. Potential participants were recruited from two sources. First, cohort participants from the PLAY Spaces & Environments for Children’s Physical Activity (PLAYCE) study were invited to participate if they were not already taking part in another PLAYCE related sub-study and if they had previously indicated a willingness to be contacted about future studies (*n* = 587 invited) [20]. The PLAYCE study, conducted from 2015–2018, investigated early childhood education and care, home, and neighbourhood environment influences on pre-schoolers’ PA [20]. Parents in this study mostly had a partner (89%), had a postgraduate education (56%), and were in either fulltime or part-time employment (81%) [21]. Second, participants were also recruited from the general community using social media strategies through Facebook posts and word of mouth. Consent was provided by a total of 170 parents.

Parents completed an online survey which assessed measures for two time points: retrospectively in February 2020 (i.e., before COVID-19 restrictions began); and in May 2020 (i.e., while COVID-19 restrictions were still in place). The online survey was developed using the Qualtrics platform and disseminated using personalised (for PLAYCE cohort recruitment) or anonymous (for general community recruitment) survey links. For participants recruited through the PLAYCE cohort, two reminders sent by email and SMS were sent to encourage survey completion. If a family had more than one child aged 5 to 9 years, parents were instructed to complete the survey for the child who celebrated their birthday more recently. An open-ended question at the end of the survey allowed respondents to provide further details on their child’s PA and wellbeing before and/or during COVID-19 distancing. Participation in this study was voluntary and all data were processed anonymously.

### 2.2. Measures

Existing validated measures of children’s PA and other movement-related behaviours (leisure screen time and sleep) were included in the online survey. In addition, parent and child demographic factors, social factors, and home environment factors were assessed.

#### 2.2.1. Physical Activity

Parents reported the frequency and duration per week their child spent doing unstructured and organised physical activities. This was reported for the two time points. These were based on existing items from the PLAYCE study’s parent-report surveys [20], which were adapted from the Healthy Active Preschool Years Study [22]. The reliability of these items is sound (e.g., unstructured physical activity items intraclass correlation (ICC) = 0.63; organised physical activity items ICC = 0.70) [22]. Additional items further assessed the percentage of time spent doing these activities at home in order to capture home-based unstructured and organised PA. Outlier responses were truncated at 14 times (frequency) and 14 h (for duration) during data processing, and measures of home-based PA computed. A measure of total PA minutes per week was computed by summing responses for duration per week of unstructured and organised PA.

Outdoor and indoor play time were measured for both time points using a slightly adapted, validated, established tool where parents reported the amount of time (five response categories: 0 min; 1–15 min; 16–30 min; 31–60 min; and >60 min) across three periods of the day (wake-up time until noon; noon until 6 pm; 6 pm until bedtime) on weekdays and weekend days that their child spent playing in the yard or street around the house; at a park, playground, or outdoor recreational area; and actively indoors at home [23]. These items have previously been validated against young children’s accelerometer data (r = 0.33, *p* < 0.001) [23]. Responses from the five response categories were coded as 0 through 4, summed across the three time periods, and averaged for week and weekend days, to give a maximum score of 12 for the three play measures: outdoor play in the yard or street around the house; outdoor play in a park, playground, or outdoor recreation area; and active indoor play at home.

#### 2.2.2. Other Movement Behaviours

Hours and minutes of sleep during the night and during the day were reported before and during COVID-19 restrictions [24]. A measure of minutes per day of sleep was computed.

Parents reported the total time their child participated in five screen-based leisure activities on weekdays and weekend days for two time points [25]. These included time spent watching television, DVDs, and online videos; using a computer (desktop or laptop) in their free time; using a tablet computer (e.g., iPad, Samsung Galaxy Tab); smartphone (e.g., iPhone, Samsung Galaxy); and games consoles—standard (e.g., PlayStation, Nintendo, X-box, Game boy, Switch). Responses were truncated and a summary measure of leisure-based screen time (minutes per week) computed for before and during COVID-19 restrictions. The total weekly screen-based entertainment score has been shown to have acceptable reliability (ICC = 0.68) [22]. The reliability for the HomeSPACE audit tool was excellent (ICC ≥ 0.80) [26].

#### 2.2.3. Demographic Factors

Parent’s reported their child’s date of birth (categorised as “5–6 years” and “7–9 years”) and sex. Parent demographic factors included: year of birth (categorised as “under 39 years” and “40 plus years”); gender; highest level of education completed (categorised as “Year 12 or less, TAFE or trade certificate” and “Bachelor degree or higher”); marital status (categorised as “in a relationship” and “no longer in a relationship or single”); work status (i.e., fulltime work, part-time work, home duties or other); and change in work status (response categorised as “yes” or “no” to the question “Has your employment status changed since the 1st of March?”) in response to COVID-19.

#### 2.2.4. Social Factors

The impact of COVID-19 on school attendance was measured by asking the number of weeks children were home-schooled during school teaching terms prior to and during COVID-19 restrictions. In 2020, term one teaching dates for primary schools were between 3 February and 9 April, and term two teaching dates were between 28 April and 3 July. Responses were used to create a disruption to school attendance variable with three response options: “minimal (i.e., none, continued attending school)”; “some (i.e., disruption in term one or two)”; and “more (i.e., disruption in both terms one and two)”.

Parents reported the ages in years and months of all children under the age of 18 years living in the household, which was used to compute a measure of number of siblings (“none”; “one sibling”; “two or more siblings”).

Number of dogs in the home was used to compute a dichotomous dog ownership variable [27].

#### 2.2.5. Home Environment Factors

Supportiveness of the home environment for active play was measured using nine modified items from the HomeSPACE tool [26]. Items included “There is enough space for my child to play actively”; “My child is able to play actively whenever he/she wants to”; and “It is safe for my child to play actively” for three home areas (inside; back yard; front yard). Parents responded using a five-point scale from strongly disagree to strongly agree. Mean scores were computed and dichotomised as “less supportive” and “more supportive” for each area of the home.

Home dwelling type was collected using an established item [28].

### 2.3. Analysis

Duplicate participants and participants missing all survey item responses were excluded, leaving an analytic sample of 157 participants. The analytic sample included even proportions of community participants recruited through Facebook (50%; 19% conversion rate) and PLAYCE cohort participants (50%; 13% response rate).

Paired *t*-tests for complete cases assessed changes in ten PA, screen time, and sleep outcomes: total PA (weekly duration); unstructured PA (weekly frequency, total duration, home-based duration); organised PA (weekly frequency, total duration, home-based duration); outdoor play in yard or street around house (weekly duration); outdoor play in park or playground or outdoor recreation area (weekly duration); active indoor play at home (weekly duration); leisure screen time (weekly duration); and sleep (daily duration). Due to the large number of statistical tests used, the Bonferroni method was used to reduce the likelihood of type 1 error and α < 0.05/10 = 0.005 was used to infer significance.

A series of mixed between-within subject’s ANOVA models were fitted to examine if changes in PA and other movement behaviours differed according to demographic (child’s age group, child’s sex, parent’s age group, parent’s highest level of education), social (disruption in school attendance, number of siblings, family dog ownership) and home environment (inside home supportiveness for PA, backyard supportiveness for PA, front yard supportiveness for PA) factors. Where significant interactions with time were found (*p* < 0.001 with Bonferroni adjustment), plots of estimated marginal means were created and stratified paired *t*-tests were run to confirm significant associations. All statistical analyses were performed using IBM SPSS Statistics for Windows, version 26 (IBM Corp., Armonk, NY, USA).

Responses to the open-ended question on the impact of COVID-19 on children’s PA and wellbeing were grouped by key themes following a coding framework. Two researchers independently coded themes, with discrepancies discussed and agreed upon together [29].

## 3. Results

### 3.1. Sample Characteristics

On average, children were 6.9 years of age (SD = 1.7). Parents were mostly highly-educated women with a bachelor’s degree or higher (75%) and working part-time (52%) (Table 1). Most children had some disruption in school attendance in either school term one or two (55%) and lived in separate house dwellings (84%).

### 3.2. Change in PA, Screen Time, and Sleep before to during COVID-19 Distancing

Overall, weekly minutes of total PA did not change from before to during COVID-19 (Table 2). However, frequency and duration (total and home-based) of unstructured PA significantly increased from before to during COVID-19 distancing. Outdoor play in the yard or street around the house, outdoor play in the park or playground or outdoor recreation area, and active indoor play at home all significantly increased from before to during COVID-19 distancing, with the greatest percentage difference found for weekly minutes of outdoor play in the park or playground or outdoor recreation area (95% increase). While frequency and total duration of organised PA significantly declined during COVID-19 distancing, a small increase of 28 min/week in home-based organised PA was not statistically significant. Compared with the time period before COVID-19 distancing, there was a significant increase in leisure screen time during COVID-19 distancing, with an additional 400 min per week reported. No sleep differences were observed.

### 3.3. Changes in PA before to during COVID-19 by Demographic, Social, and Home Environment Factors

With Bonferroni adjustment, no significant interactions with time were found at *p* < 0.001.

A summary of key themes to emerge from the thematic analysis of open-ended responses is presented in Table 3. Parents identified that not having to commute to work meant there was time available to support their children actively travel (e.g., ride or walk) to and from school, and that it was an opportunity to spend more time together as a family. Parents reported their children were more physically active during COVID-19 distancing and this was due to more children playing with each other in local streets; increased use of neighbourhood spaces (e.g., parks) for PA; increased unstructured physical activities as a result of organised sport being forced to stop; and parents’ direct attempts to manage family mental health challenges through increased PA.

## 4. Discussion

During Western Australian COVID-19 restrictions, there was no change in overall PA among children, yet a compensation effect whereby an increase in young children’s unstructured PA, outdoor play and leisure screen time, and a decrease in organised PA was found. Qualitative findings showed the importance of a variety of demographic, social, and home environment factors impacted on changes in children’s PA behaviours during this period of physical distancing restrictions. While these findings present a “good news” story in terms of the impact of COVID-19 restrictions on young children’s PA, they also highlight the adaptive nature of children and families to a changing environment and life imposed by these restrictions. COVID-19 restrictions prevented any group-based organised PA, yet parents and children compensated for this through increasing their time spent doing unstructured PA at home (outdoors and indoors) and at local parks. Our findings highlight the innate need for young children to play, be outdoors and active and the need to afford them more time to do this on a daily basis through multilevel intervention approaches.

To date, only three studies have quantitatively reported the impact of COVID-19 on children’s PA behaviour [14,15,16]. In a Canadian survey of parents of 693 children aged 5–11 years, PA levels and time spent outside declined, while leisure screen time and sleep was higher during the outbreak compared to before ^15^. These findings are in contrast to our own, which in part may be explained by methodological differences between studies. For example, our study included a smaller age range of young children 5–9 years, and we specifically asked about organised and unstructured PA as well as outdoor and indoor play, while the Canadian study focused on specific moderate–vigorous physical activities. Comparing findings is difficult, as the Canadian study provided little detail of the survey timing in relation to the onset and duration of COVID-19 restrictions in Canada. Restrictions also likely varied across different geographical areas influencing parents response to whether their child’s PA behaviour was “a lot less, a little less, the same, a little more, or a lot more” compared to before the COVID-19 outbreak and related restrictions [15]. The differences in findings may also have been due to the pleasant weather patterns experienced in Western Australia. In May 2020, the average maximum temperature was 22 degrees Celsius in Perth [30]. Nevertheless, our findings are consistent with preliminary evidence indicating an increase in Australian adults interest in being active during the COVID-19 pandemic when physical distancing restrictions were in place [31]. Overall, these findings highlight the need for family-level PA interventions to minimise any negative impact of COVID-19 restrictions on children’s PA levels.

Overall, the increases in young children’s unstructured PA, outdoor play, and active indoor play we observed between pre- and during COVID-19 restrictions highlights that children (and parents) may have been replacing time lost in organised PA with more unstructured play-based activities. Active play is critical not only for children’s physical and mental health but facilities a child’s cognitive and social–emotional development [32]. Organised PA, such as team sports, is a critical way for children to develop socially [33] as well as develop their fundamental movement skills [34,35]. A more sustained stop to all group sports and physical activities because of COVID-19 restrictions could have a long-term negative impact on children’s physical development, and ability and confidence to participate in PA throughout childhood and into adult life [2]. This is due to the popularity of organised sport and physical activity participation (especially in school) among young Australian children [3]. Despite the observed declines in organised PA, parents also commented on how families were using environments differently to keep active through walking and cycling, which, in particular, was increasing their children’s riding confidence. Parents also described how their children were playing in the street and with their neighbours more, highlighting that children may be socialising through unstructured PA despite the lack of organised PA. It will be important for ongoing monitoring of the impact of COVID-19 on children’s PA to examine if compensation effects (i.e., a move from organised/structured to unstructured PA) is maintained and is sustainable in the longer term.

These results are in support of some preliminary evidence so far showing that Canadian, Chinese, and Korean children’s screen time increased significantly during COVID-19 restrictions [13]. This highlights the need for resources and strategies to better support children and families to best manage screen time (for leisure) during, post, and in potential subsequent waves of the COVID-19 pandemic.

Our findings make sense in the Western Australian context of a relatively short COVID-19 lockdown period, but also provide preliminary directions for implications and interventions in the future. First, our results suggest the benefits of having “exercise permitted” restrictions (but not group physical activities) amongst a suite of physical distancing restrictions. In Australia, government officials provided consistent messaging that “exercise” was a permitted reason to leave the house. Indeed, research shows that mass-media campaigns with passive exposure to health-promoting messaging can produce positive changes in health-related behaviours across large populations [36]. The consistent messaging by the Australian Government on exercise during COVID-19 restrictions afforded families with young children the opportunity to get outside and visit the local park (albeit the use of playground structures was not permitted). Most children also had relatively supportive home environments to enable more unstructured PA both indoors and outdoors. This is due to the low-density housing in Perth, Western Australia, with many families choosing to live in a larger, family-size house with a backyard [37]. Another implication of this study’s findings is the importance of supporting families, in particular parents, around guidance on how they can facilitate children’s unstructured PA. Beyond pandemics, families are an important influence on children’s PA behaviour [38,39,40]. Adapting existing resources to support parents in providing more PA opportunities for their children at home is a feasible intervention that can be undertaken should subsequent COVID-19 waves occur (see the KIDDO program resources as an example (https://kiddo.edu.au/) accessed on 1 March 2021). These implications are all consistent with socioecological models of health behaviour, further highlighting the importance of multilevel interventions to change children’s PA behaviour. However, it is imperative that measures are considered within the context of other physical distancing restrictions in place to reduce the risk of community transmission (e.g., border controls). In Western Australia, while families were permitted to be active within the neighbourhood, this was only possible because of the reduced risk of virus transmission within the community due to strict border controls and quarantine arrangements which were operating at the time.

This study was limited by its small sample size which compromised statistical power. Further statistical limitations include the number of statistical tests conducted, the large standard deviations, and large differences in cell sizes limiting the robustness of the results. In addition, results were self-reported by parents, mostly mothers who were more highly-educated than the general population in Australia [41], potentially limiting the generalizability of the study’s findings. Given the context-specific nature of the pandemic and the imposed restrictions within and between countries, the findings may not be generalisable to other Australian states and territories or countries. Nevertheless, the context specificity of the timing, location, and design of the study to measure the impact of before and during COVID-19 restrictions on Western Australian children’s PA behaviour is a strength. Finally, parents were highly educated, and thus further research is needed with vulnerable families.

## 5. Conclusions

Overall, the findings highlight the adaptive nature of children and families in light of the COVID-19 pandemic, with observed increases in children’s unstructured PA replacing the observed decreases in organised PA. It remains to be seen whether children’s increased unstructured PA and outdoor play have been sustained now that Western Australia has had all except the last stage of restrictions lifted, or what would happen if there was a second wave of COVID-19 and restrictions were reimplemented, as has occurred in other Australian states and countries. The impact of COVID-19 on children’s PA is likely to be specific to the severity and length of each COVID-19 outbreak on a particular geographical population (and its sub-groups), as well as the geographical or other specific restrictions that were enacted, the time they were in place, and how quickly they were lifted. Further context-specific research is required to better inform the guidance and supports required to ensure children’s overall PA is not negatively impacted by COVID-19 and other potential future pandemics or natural disasters.

## Figures and Tables

**Table 1:** Sample characteristics (*n* = 157).

**Child Demographic Factors**	***n***	**%**
Age group		
5–6 years	48	30.6
7–9 years	109	69.4
Sex		
Boy	85	54.1
Girl	72	45.9
**Parent demographic factors**		
Age group ^1^		
Under 39 years	75	48.1
40+ years	81	51.9
Gender		
Male	7	4.5
Female	149	94.9
Other	1	0.6
Highest level of education		
Years 12 or less, TAFE or Trade Certificate	40	25.5
Bachelor degree or higher	117	74.5
Marital status ^2^		
In a relationship	91	90.1
No longer in a relationship or single	10	9.9
Work status ^2^		
Full-time work	27	26.7
Part-time work	52	51.5
Home duties	12	11.9
Other	10	9.9
Work status change due to COVID-19 ^2^		
No change	81	80.2
No longer employed	8	7.9
Decrease in work hours	11	10.9
Increase in work hours	1	1.0
**Social factors**		
Disruption in school attendance ^3^		
Minimal (i.e., none, continued attending school)	18	16.1
Some (i.e., disruption in term one or two)	61	54.5
More (i.e., disruption in both terms one and two)	33	29.5
Number of siblings ^2^		
None	19	18.8
One sibling	58	57.4
Two or more siblings	24	23.8
Dog ownership ^4^		
Non-owner	66	57.4
Dog owner	49	42.6
**Home environment factors**		
Inside home supportiveness for PA ^2^		
Less supportive	43	42.6
More supportive	58	57.4
Backyard supportiveness for PA ^2^		
Less supportive	54	53.5
More supportive	47	46.5
Front yard supportiveness for PA ^2^		
Less supportive	68	67.3
More supportive	33	32.7
Type of dwelling ^5^		
Separate house	86	84.3
Semi-detached house or duplex	2	2.0
Townhouse or terrace house	3	2.9
Single story flat or home unit	7	6.9
Flat or unit in block of 2 or 3 storeys	2	2.0
House or flat attached to office, shop, etc.	1	1.0
Other	1	1.0

^1^*n* = 1 missing. ^2^ *n* = 56 missing. ^3^ *n* = 45 missing. ^4^ *n* = 42 missing. ^5^ *n* = 55 missing.

**Table 2:** Change in movement behaviours from before to during COVID-19 distancing.

	*n*	Before COVID-19 Distancing		During COVID-19 Distancing		Difference	Percentage Difference	*p*
		Mean	SD	Mean	SD			
Total PA (min/week)	121	809.7	584.4	835.4	642.4	25.7	3.2	0.647
Unstructured PA (times/week)	122	6.0	3.0	7.8	4.3	1.8	30.0	<0.001
Unstructured PA (min/week)	120	632.3	540.3	778.6	606.5	146.3	23.1	0.005
Home-based unstructured PA (min/week)	113	342.3	408.5	543.3	504.5	201.0	58.7	<0.001
Organised PA (times/week)	112	2.0	1.3	0.7	1.8	−1.3	−65.0	<0.001
Organised PA (min/week)	99	189.7	178.5	65.1	170.6	−124.6	−65.7	<0.001
Home-based organised PA (min/week)	98	16.1	45.4	43.6	135.3	27.5	170.8	0.051
Outdoor play in yard or street around house (score)	118	5.4	2.0	6.1	2.3	0.7	12.9	<0.001
Outdoor play in park or playground or outdoor recreation area (score)	117	1.9	1.8	3.7	2.3	1.8	94.7	<0.001
Active indoor play at home (score)	116	6.0	3.0	7.9	2.9	1.9	31.7	<0.001
Leisure screen time (min/week)	101	794.2	565.5	1194.2	843.5	400.0	50.4	<0.001
Sleep (mins/day)	112	614.8	48.5	612.8	67.0	−2.0	−0.3	0.639

**Table 3:** Summary of key themes identified from open-ended responses on children’s physical activity (PA) and wellbeing before and/or during COVID-19 distancing.

Theme	Description	Example Quotes
Organised Sport	Parents discussed impact of changes in structured sporting activities on their children.	*“The only thing they missed out on during COVID-19 distancing was their ice skating lessons which we couldn’t obviously continue at home, so I went and bought them inline skates to make up for it.”*
	Some families enjoyed the unstructured time they gained and other families missed the connection and structure these activities provided.	*“My oldest child is very sporty and does prefer structured activity, so keeping her active and moving during COVID-19 distancing was harder than expected. She is looking forward to sports starting back up.”*
Sibling Relationships	Families described strengthening of the sibling relationships and the positive aspects of their children playing together.	*“Both our children were relaxed and played well with each other and I think bonded a lot more over the weeks we were at home.”*
Technology use	Parents spoke of the challenges and benefits of increased technology use during this time.	*“The most significant challenge we face on a daily/by hour basis is managing our kids screen time and screen content.”*
	Some families struggled with increased technology use. Others acknowledged that it had increased but could be positive, such as educational or PA-promoting, and helped to maintain connection with extended family and friends.	*“Lots more screen time—some for educational purposes. However, they are connecting more often with interstate family as everyone is doing more virtual connecting rather than physical connections. So that’s a positive that my kids feel closer to extended family and friends on the east coast.”*
Neighbourhood connection	Some families spoke of quieter streets and increased levels of isolation. Other families spoke about getting to know their neighbours for the first time and everyone’s kids playing together on the streets and the increased neighbourhood connection.	*“COVID-19 distancing has been challenging, but the one area that has blossomed has been neighbourhood connection and freedom to just hang out in the street together. It’s been a huge positive side effect and one I’ve desired for years.”*
Pets	Chickens and dogs encouraged families to get outside and move together. Some families reported that their children became closer to their pets and had a positive impact on their mental health.	*“We had gotten a puppy back in December 2019 and during the isolation period the kids started walking him daily with us as part of our routine.”*
Adults work life balance	Some families mentioned increased screen time and the stress associated with taking work conference calls and background noise. Several families reported the positive aspects of more unstructured time together outdoors and being active and the strengthening of the family relationships.	*“Since going back to school and changing my work hours, we now walk to school every day and this is a new schedule here to stay. The silver lining as far as I am concerned. We also walk more as a family on the weekend now too.”*
Built environment	Parents discussed how they were using their environments differently with access to schools, playgrounds, and organised sport restricted.	*“Playgrounds have always been a big part of our life as we don’t have much of a backyard, so we’ve had to move our play to the front yard where we play tennis, cricket, footy* etc. *Bike rides around the neighbourhood have now become a daily activity where they used to be only a weekend or occasional activity.”*
	Many families mentioned increases in bike rides, exploring the neighbourhood on a daily rather than weekend basis and increases in their children’s confidence riding. Some families discussed the challenges having a small yard or no fence posed.	*“As a family we have probably been more active, going on bike rides, kayaking and long walks.”*

## Data Availability

The non-identifiable data presented in this study are available on reasonable request from the corresponding author.

## References

[1] Janessen I., LeBlanc A. (2010). Systematic review of the health benefits of physical activity and fitness in school-aged children and youth. Int. J. Behav. Nutr. Phys. Act..

[2] Jones R.A., Hinkley T., Okely A.D., Salmon J. (2013). Tracking physical activity and sedentary behavior in childhood: A systematic review. Am. J. Prev. Med..

[3] Active Healthy Kids Australia (2018). Muscular Fitness: It’s Time for a Jump Start. The 2018 Active Healthy Kids Australia Report Card on Physical Activity for Children and Young People.

[4] Spink K., Shields C., Chad K., Odnokon P., Muhararine N., Humbert L. (2006). Correlates of structured and unstructured activity among sufficiently active youth and adolescents: A new approach to understanding physical activity. Ped. Exerc. Sci..

[5] Witten K., Kearns R., Carroll P., Asiasiga L., Tava’e N. (2013). New Zealand parents’ understandings of the intergenerational decline in children’s independent outdoor play and active travel. Child Geogr..

[6] Tremblay M.S., Carson V., Chaput J.-P., Connor Gorber S., Dinh T., Duggan M., Faulkner G., Gray C.E., Gruber R., Janson K. (2016). Canadian 24-hour movement guidelines for children and youth: An integration of physical activity, sedentary behaviour, and sleep. Appl. Physiol. Nutr. Metab..

[7] Australian Government Department of Health (2017). Australian 24—Hour Movement Guidelines for the Early Years (Birth to 5 Years): An Integration of Physical Activity, Sedentary Behaviour, and Sleep.

[8] World Health Organization (2019). Guidelines on Physical Activity, Sedentary Behaviour and Sleep for Children under 5 Years of Age: Web Annex: Summary.

[9] Stokols D. (1992). Establishing and maintaining healthy environments: Toward a social ecology of health promotion. Am. Psychol..

[10] Sallis J., Prochaska J., Taylor W. (2000). A review of correlates of physical activity of children and adolescents. Med. Sci. Sports Exerc..

[11] World Health Organization Rolling Updates on Coronavirus Disease (COVID-19). https://www.who.int/emergencies/diseases/novel-coronavirus-2019/events-as-they-happen.

[12] Government of Western Australia Media Statements. https://www.mediastatements.wa.gov.au/Pages/Ministers/Mark-McGowan.aspx.

[13] Guan H., Okely A.D., Aguilar-Farias N., del Pozo Cruz B., Draper C.E., El Hamdouchi A., Florindo A.A., Jáuregui A., Katzmarzyk P.T., Kontsevaya A. (2020). Promoting healthy movement behaviours among children during the COVID-19 pandemic. Lancet Child Adolesc. Health.

[14] Dunton G.F., Do B., Wang S.D. (2020). Early effects of the COVID-19 pandemic on physical activity and sedentary behavior in children living in the U.S. BMC Public Health.

[15] Moore S.A., Faulkner G., Rhodes R.E., Brussoni M., Chulak-Bozzer T., Ferguson L.J., Mitra R., O’Reilly N., Spence J.C., Vanderloo L.M. (2020). Impact of the COVID-19 virus outbreak on movement and play behaviours of Canadian children and youth: A national survey. Int. J Behav. Nutr. Phys. Act..

[16] Xiang M., Zhang Z., Kuwahara K. (2020). Impact of COVID-19 pandemic on children and adolescents’ lifestyle behavior larger than expected. Prog. Cardiovasc. Dis..

[17] WA Department of the Premier and Cabinet COVID-19 Coronavirus. https://www.wa.gov.au/government/covid-19-coronavirus.

[18] ABS (2018). 3218.0—Regional Population Growth, Australia, 2016–2017.

[19] De Vet E., De Ridder D., De Wit J. (2011). Environmental correlates of physical activity and dietary behaviours among young people: A systematic review of reviews. Obes. Rev..

[20] Christian H., Maitland C., Enkel S., Trapp G., Trost S.G., Schipperijn J., Boruff B., Lester L., Rosenberg M., Zubrick S.R. (2016). Influence of the day care, home and neighbourhood environment on young children’s physical activity and health: Protocol for the PLAYCE observational study. BMJ Open.

[21] Wenden E.J., Lester L., Zubrick S.R., Ng M., Christian H.E. (2020). The relationship between dog ownership, dog play, family dog walking, and pre-schooler social–emotional development: Findings from the PLAYCE observational study. Pediatric Res..

[22] Hinkley T., Salmon J., Okely A.D., Crawford D., Hesketh K. (2012). The HAPPY Study: Development and reliability of a parent survey to assess correlates of preschool children’s physical activity. J. Sci. Med. Sport.

[23] Burdette H.L., Whitaker R.C., Daniels S.R. (2004). Parental report of outdoor playtime as a measure of physical activity in preschool-aged children. Arch. Pediatr. Adolesc. Med..

[24] Australian Government Department of Social Services Growing up in Australia: The Longitudinal Study of Australian Children Survey. https://www.dss.gov.au/about-the-department/publications-articles/research-publications/longitudinal-data-initiatives/footprints-in-time-the-longitudinal-study-of-indigenous-children-lsic/growing-up-in-australia-the-longitudinal-study-of-australian-children-lsac.

[25] Hinkley T., O’Connell E., Okely A.D., Crawford D., Hesketh K., Salmon J. (2012). Assessing volume of accelerometry data for reliability in preschool children. Med. Sci. Sports Exerc..

[26] Maitland C., Foster S., Stratton G., Braham R., Rosenberg M. (2019). Capturing the geography of children’s active and sedentary behaviours at home: The HomeSPACE measurement tool. Child Geogr..

[27] Cutt H., Giles-Corti B., Knuiman M., Pikora T. (2008). Physical activity behavior of dog owners: Development and reliability of the Dogs And Physical Activity (DAPA) tool. J. Phys. Act. Health.

[28] ABS (2007). 2914.0- 2006 Census of Population and Housing.

[29] Creswell J.W., Creswell J.D. (2018). Research Design: Qualitative, Quantitative, and Mixed methods Approaches.

[30] Commonwealth of Australia, Bureau of Meteorology (2020). Perth, Western Australia, May 2020 Daily Weather Observations.

[31] Ding D., del Pozo Cruz B., Green M.A., Bauman A.E. (2020). Is the COVID-19 lockdown nudging people to be more active: A big data analysis. Br. J. Sports Med..

[32] Carson V., Lee E.-Y., Hewitt L., Jennings C., Hunter S., Kuzik N., Stearns J.A., Unrau S.P., Poitras V.J., Gray C. (2017). Systematic review of the relationships between physical activity and health indicators in the early years (0–4 years). BMC Public Health.

[33] Eime R.M., Young J.A., Harvey J.T., Charity M.J., Payne W.R. (2013). A systematic review of the psychological and social benefits of participation in sport for children and adolescents: Informing development of a conceptual model of health through sport. Int. J. Behav Nutr. Phys. Act..

[34] Lopes V.P., Rodrigues L.P., Maia J.A., Malina R.M. (2011). Motor coordination as predictor of physical activity in childhood. Scand J. Med. Sci. Sports.

[35] Barnett L.M., Van Beurden E., Morgan P.J., Brooks L.O., Beard J.R. (2009). Childhood motor skill proficiency as a predictor of adolescent physical activity. J. Adolesc. Health.

[36] Wakefield M.A.P., Loken B.P., Hornik R.C.P. (2010). Use of mass media campaigns to change health behaviour. Lancet.

[37] Roberts B.H. Changes in urban density: Its implications on the sustainable development of Australian cities. Proceedings of the State of Australian Cities National Conference.

[38] Korcz A., Krzysztoszek J., Łopatka M., Ludwiczak M., Górska P., Bronikowski M. (2020). The role of family time together in meeting the recommendation for physical activity among primary school children. Int. J. Environ. Res. Public Health.

[39] Pluta B., Bronikowska M., Tomczak M., Laudańska-Krzemińska I., Bronikowski M. (2017). Family leisure-time physical activities–results of the “Juniors for Seniors” 15-week intervention programme. Biomed. Hum. Kinet..

[40] Bronikowski M., Bronikowska M., Pluta B., Maciaszek J., Tomczak M., Glapa A. (2016). Positive impact on physical activity and health behaviour changes of a 15-week family focused intervention program: “Juniors for Seniors”. BioMed Res. Int..

[41] ABS (2017). 2900.0—Census of Population and Housing: Understanding the Census and Census Data, Australia, 2016.

